# The leptospiral LipL21 and LipL41 proteins exhibit a broad spectrum of interactions with host cell components

**DOI:** 10.1080/21505594.2021.1993427

**Published:** 2021-11-25

**Authors:** Takahashi MB, Teixeira AF, Nascimento ALTO

**Affiliations:** a Laboratório de Desenvolvimento de Vacinas, Instituto Butantan, São Paulo, Brazil; b Programa de Pós-Graduação Interunidades Em Biotecnologia, Instituto de Ciências Biomédicas, Universidade de São Paulo, São Paulo, Brazil

**Keywords:** *Leptospira* spp, leptospirosis, pathogenesis, LipL21, LipL41

## Abstract

Leptospirosis is a globally prevalent zoonotic disease, and is caused by pathogenic spirochetes from the genus *Leptospira*. LipL21 and LipL41 are lipoproteins expressed strongly on the outer membrane of pathogenic *Leptospira* spp. Many studies have shown that both proteins are interesting targets for vaccines and diagnosis. However, their role in host–pathogen interactions remains underexplored. Therefore, we evaluated the capacity of LipL21 and LipL41 to bind with glycosaminoglycans (GAGs), the cell receptors and extracellular matrix, and plasma components by ELISA. Both proteins interacted with collagen IV, laminin, E-cadherin, and elastin dose-dependently. A broad-spectrum binding to plasma components was also observed. Only LipL21 interacted with all the GAG components tested, whereas LipL41 presented a concentration-dependent binding only for chondroitin 4 sulfate. Although, both proteins have the ability to interact with fibrinogen, only LipL21 inhibited fibrin clot formation partially. Both proteins exhibited a decrease in plasminogen binding in the presence of amino caproic acid (ACA), a competitive inhibitor of lysine residues, suggesting that their binding occurs via the kringle domains of plasminogen. LipL41, but not LipL21, was able to convert plasminogen to plasmin, and recruit plasminogen from normal human serum, suggesting that the interaction of this protein with plasminogen may occur in physiological conditions. This work provides the first report demonstrating the capacity of LipL21 and LipL41 to interact with a broad range of host components, highlighting their importance in host–*Leptospira* interactions.

## Introduction

Pathogenic *Leptospira* is the etiological agent of leptospirosis, an important zoonotic disease prevalent worldwide. The infection occurs through the host’s mucosa or injured skin directly after contact with urine of infected animals, or indirectly after exposure to contaminated water or soil. Initial appearance of mild flu-like symptoms, such as fever, headache, and nausea, may lead to an inaccurate diagnosis (i.e., dengue, influenza, or yellow fever). However, the disease may progress to severity, resulting in pulmonary hemorrhage, renal and hepatic failure, and Weil’s disease, with a mortality rate of up to 50% [1–3].

Host–pathogen interactions in *Leptospira* spp. are poorly understood. Elucidation of pathogenic molecular mechanisms can aid the development of more effective vaccines and efficient diagnostic and treatment strategies. Outer membrane proteins are considered the main candidates for vaccines and diagnosis, as they are the first proteins to interact with the host and promote an immune response. Major outer membrane proteins of pathogenic leptospires include LipL32, Loa22, LipL41, LipL36, and LipL21 [4]. LipL32, a 32 kDa protein with approximately 38,000 copies per cell in *L. interrogans*, is the most abundantly expressed outer membrane protein in pathogenic strains [5], and the most extensively studied leptospiral protein [6–11].

LipL41 is a 41 kDa lipoprotein, and was first identified as a major component in the detergent phase following Triton X-114 solubilization and partitioning of *L. kirschneri* [12]. Lipidation and lipoprotein signal peptide of this protein were verified by incorporating radiolabelled palmitate and inhibiting protein processing by globomycin, which inhibits lipoprotein signal peptidases selectively [13]. LipL41 is co-transcribed with a small chaperone called *lep*, essential for protein stability during expression, and forms an oligomer containing 36 units that folds as a double-layered particle [14,15]. LipL21, a 21 kDa protein, is the second most abundantly expressed outer membrane protein isolated from *L. interrogans* serovar Lai; it has been identified as a lipoprotein by tritiated palmitic acid assay, and was isolated from the detergent phase following Triton X-114 membrane fractioning [16]. There is strong evidence indicating that LipL41 and LipL21 have surface localization, making them important targets for the induction of host immune response [4].

It has been shown that LipL32 has the ability to bind with several host components, such as laminin, collagen types I, V, IV, and XX, fibrinogen, and plasminogen [17]. Moreover, its capacity to bind with HUVEC cells *in vitro* and increase cell permeability has also been shown [18]. Loa22 has been shown to interact with several chondroitin sulfate-type proteoglycans [19]. Despite the fact that LipL21 and LipL41 are major outer membrane proteins of, and are expressed only in pathogenic leptospires, their role in pathogenesis is yet to be investigated.

In this study, we aimed to investigate the role of LipL21 and LipL41 in host interaction, with emphasis on binding with the extracellular matrix (ECM) molecules, plasma proteins, and glycosaminoglycans (GAGs). Thus, LipL21 and LipL41 coding sequences were cloned, and the proteins were expressed and purified for interaction analysis with host components.

## Materials and methods

### Bioinformatics analysis

Coding sequences (CDs) of *L. interrogans* serovar Copenhageni str. Fiocruz L1-130 for LipL21 (LIC10011) and LipL41 (LIC12966) proteins deposited in the NCBI database were compared using NCBI blastp [20], PFAM [21], and SMART [22] to identify the conserved domains. Identification of similarity for LipL21 and LipL41 from *L. interrogans* serovar Copenhageni among others leptospires was performed by Clustal Omega software [23]. Protein tertiary structures were modeled by I-TASSER based on the similarity of structures [24]. The C-score, which is based on the significance of threading template alignments and the convergence parameters of the structure assembly simulations, was used as parameter to quantify the confidence by the I-TASSER software. This value usually varies from [−5,2] and the highest value means higher confidence.

### Cloning, expression, and purification of recombinant proteins

*L. interrogans* serovar Copenhageni strain M20 genomic DNA was used as a template for amplifying LipL21 and LipL41 genes. Two sets of primers were designed as follows: forward 5′-CGCGAATTCTCCAGTACTGACACAGGA-3′ (EcoRI), and reverse 5′-GGCGCTCGAGTTATTGTTTGGAAACCTC-3′ (XhoI) for LipL21; and forward 5′-GCG**GAATTC**GCAGCTACAGTCGATGTAGAA-3′ (EcoRI), and reverse 5′-CGCG**CTCGAG**TTACTTTGCGTTGCTTTCATC-3′ (XhoI) for LipL41. Restriction sites EcoRI and XhoI were added to the oligonucleotides to clone the sequences into the pET28a-SUMO (Small ubiquitin-like modifier) vector [25]. This vector adds a fusion tag containing six histidine residues followed by a SUMO at the N-terminus. Amplification was performed without the predicted signal peptide region, which was analyzed by the software LipoP [26]. After PCR amplification, the reaction products were purified using a GFX™ PCR DNA and Gel Band Purification kit (GE Healthcare, Chicago, Illinois, USA), and cloned into pET28a-SUMO at the restriction sites. The constructs were analyzed by DNA sequencing using the primers T7 (forward: 5′-TAATACGACTCACTATAGGG-3′) and T7R (reverse: 5′-TAGTTATTGCTCAGCGGTGG-3′) on an ABI sequencer (PE Applied Biosystems, Foster City, California, USA). *E. coli* BL21 (DE3) strain was used as host for protein expression. A total of 400 mL of culture was inoculated with 4 mL (1%) of an overnight growth culture, and incubated at 37°C until an optical density (OD_600nm_) of 0.6 was achieved. Subsequently, the culture was induced for 3 h at 37°C with 1 mM IPTG. Bacteria were harvested by centrifugation, resuspended in 30 mL sonication buffer (10 mM Tris [pH 8.0], 150 mM NaCl, 200 μg/mL lysozyme, 2 mM phenylmethylsulphonyl fluoride [PMSF], and 1% Triton X-100), and sonicated for 10 min on ice. The soluble fraction was obtained from the supernatant of the cell lysate by centrifugation at 12,000 × *g* for 10 min at 4°C. Purification was started with immobilized metal affinity chromatography (IMAC) with an imidazole gradient (50 mL of each concentration of imidazole were used as wash steps: 5, 20, 40, and 60 mM imidazole, 20 mM Tris [pH 8.0], 500 mM NaCl), and elution was performed with 15 mL of a solution containing 500 mM imidazole, 20 mM Tris (pH 8.0), and 500 mM NaCl to recover the recombinant protein. Next, a SUMO protease (Ubiquitin-like-specific protease – Ulp1), which recognizes the SUMO C-terminal, was added at a proportion of 1:100 (SUMO: recombinant protein in relation to their mass) to cleave the fusion tag from the target protein, for 16 h at 4°C. A dialysis step was carried out against phosphate buffered saline (PBS, pH 7.4) to remove imidazole and exchange buffers. Finally, a second IMAC was performed to remove both SUMO and Ulp1 from the target protein using 5 mL of PBS. The recombinant leptospiral protein without the His tag was collected from the flow-through, and the fusion protein system (SUMO and Ulp1) was attached to the column. Purified proteins were quantified using a Bradford kit, and analyzed by SDS-PAGE.

### Analysis of recombinant protein secondary structure by circular dichroism (CD)

Recombinant protein samples were dialyzed against sodium phosphate buffer (10 mM, pH 7.4) for 16 h at 4°C under agitation. The analysis was performed at 20°C using a Jasco J-810 spectropolarimeter (Japan Spectroscopic, Tokyo, Japan) equipped with a Peltier unit for temperature control. Measurements were taken ten times from 190 to 250 nm at 0.5 nm intervals, using a cell with 1 mm path length. CD spectra were measured by residual molar ellipticity (Θ × L × C × 10^3^), where Θ (deg) is the ellipticity, L (cm^2^) refers to the optic path length, and C (dmol^−1^) is the protein concentration. The secondary structure of the recombinant proteins was modeled based on the mean of the experimental readings by BeStSel [27] and CAPITO [28] softwares.

### Binding of recombinant proteins to purified host receptors

Individual components of the ECM, plasma, and GAG (Sigma-Aldrich, St. Louis, Missouri, USA) were used to examine the interaction of the recombinant proteins . The assay was performed as previously described, with certain modifications [19]. ELISA plates containing 96 wells (High Binding, F; Sarstedt, Rommelsdorf, Nümbrecht, Germany) were coated with 1 µg/well of elastin (*human aorta*, F5881), collagen type I (*rat tail*, C3867, and *calf skin*, C8919), laminin (*human placenta*, L6274), cellular fibronectin (*human foreskin fibroblasts*, 2518), and E-cadherin (*human recombinant*, 5085), representing the cell receptors and ECM components. For the plasma components, the same mass was used to coat the plates using plasma fibronectin (*human plasma*, F2006), fibrinogen (*human plasma*, F4883), and plasminogen (*human plasma*, P7999), except vitronectin (*human plasma*, V8379), which was incubated at 250 ng/well. For GAG binding, wells were coated with 100 µg each of heparin (*porcine intestinal mucosa*, H4784), chondroitin sulfate (*shark cartilage*, C4384), and chondroitin 4 sulfate (*bovine trachea*, 27,042), and 1 µg of heparan sulfate (*bovine kidney*, H7640). Fetuin (*fetal bovine serum*, F3385) and BSA (*bovine serum albumin*, Bovostar; Bovogen Biologicals, Melbourne, Victoria, Australia) were used as negative controls with the addition of 1 µg/well. The statistical significance using 1 µg/well of the negative controls was similar to 100 µg/well, then, 1 µg of each control was used for all assays. The values of absorbance were deduced from the value of a control lacking the recombinant proteins in the reaction. The ELISA plates were incubated with the components for 16 h at 4°C in PBS in a final volume of 100 µL. Then, the wells were washed three times with PBS containing 0.05% Tween 20 (PBS-T). A blocking solution containing PBS with 1% BSA (PBS-BSA) was added for 2 h at 37°C in a final volume of 200 µL. Then, the recombinant proteins (1 µg/well) were incubated with the blocking solution for 2 h at 37°C in a final volume of 100 µL. The lipoprotein LipL46 produced in pET-SUMO system was used as control, as this protein is known to bind to plasminogen only [29]. For GAG analysis, wells were fixed with 2% paraformaldehyde in PBS for 30 min, followed by incubation with 2% glycine for 30 min at room temperature (reaction using 100 µL). After washing, polyclonal antibodies against each recombinant protein were incubated for 1 h at 37°C (dilution of 1:500 for LipL21, and 1:1,000 for LipL41, reaction using 100 µL). Binding detection was performed using HRP-conjugated goat anti-mouse IgG (1:5,000), followed by the addition of citrate phosphate buffer (150 mM, pH 5.0) containing 1 mg/mL of o-phenylenediamine and 0.03% H_2_O_2_ (reaction using 100 µL). After 15 min, the reaction was stopped by adding 50 µL of 2 M H_2_SO_4_, and the absorbance (OD_492nm_) was measured using a Multiskan-FC microplate reader (Thermo Fisher Scientific, Helsinki, Finland). Ligation of the recombinant proteins was compared to the negative controls by two-tailed *t*-test (p < 0.05) in GraphPad Prism software v. 7 (GraphPad Prism, San Diego, CA, USA).

### Dose-response of recombinant proteins to host components

Host components that interacted to the recombinant proteins were coated in 96-well ELISA plates as described above. After the blocking step, increasing concentrations of recombinant proteins in PBS-BSA were added to the plates for 2 h at 37°C. For the GAG dose-response assay, a paraformaldehyde fixation step, followed by glycine incubation was included, which was in turn followed by the same binding procedure. Antibody incubation and detection of reaction were performed as described above. For vitronectin, laminin, and plasminogen binding, the primary antibody against the recombinant proteins was diluted to 1:5,000. The dose-response curves and dissociation constant calculation were fitted by the tool in GraphPad Prism software v. 7 “*Non-linear regression”*, considering saturation binding with “*one site-specific binding*”. The statistical difference by t-test comparing the binding of the proteins to GAG and 1 µg of fetuin or BSA is correspondent to 100 µg of the controls. Thus, we evaluated the assays using 1 µg of each control.

### Fibrin clot inhibition assay

Two concentrations of each recombinant protein were incubated with 1 mg/mL fibrinogen in 150 mM NaCl buffer for 2 h at 37°C. Then, in a 96-well plate, 90 µL of fibrinogen solutions were incubated with 10 μL of thrombin (10 U/mL). The positive control is represented by the co-incubation of thrombin and fibrinogen and negative control is the reading of thrombin sample without the addition of fibrinogen, both without the addition of the recombinant proteins. Fibrin clot formation was measured in a microplate reader (Multiskan-FC) at OD_595nm_ for 1 h at 1 min interval.

### Inhibition of plasminogen binding by amino caproic acid (ACA)

For assaying the inhibition of plasminogen binding, 96-well ELISA plates were coated with 1 µg of plasminogen for 16 h at 4°C. The plates were washed with PBS-T, and the blocking solution was added for 2 h at 37°C. The recombinant protein was added with two different concentrations (2 and 20 mM) of ACA (Sigma-Aldrich) for 2 h at 37°C. The plate was washed with PBS-T, and the reaction was detected as described above.

### Plasminogen recruitment from normal human sera

ELISA plates were coated with recombinant proteins or BSA (1 µg/well) for 16 h at 4°C. The plates were washed with PBS-T, and the blocking solution was added for 2 h at 37°C. Then, normal human serum (Sigma-Aldrich) (0 to 20%) was added in each well. Ligation of the plasminogen to the recombinant proteins was detected by mouse anti-plasminogen (1:5,000) for 1 h at 37°C, followed by HRP-conjugated goat anti-mouse IgG (1:5,000). The reaction was detected as described above.

### Enzymatic assay of plasminogen conversion to plasmin

For the enzymatic assay of plasminogen conversion to plasmin, 96-well plates were coated with the recombinant proteins or BSA as described above. After the washing and blocking steps, plasminogen was added to the wells (1 µg/well) for 2 h at 37°C. Then, the plates were washed, and 5 ng of human urokinase plasminogen activator (uPA; Sigma-Aldrich) and 0.8 mM of plasmin-specific substrate D-valyl leucyl-lysine-p-nitroanilide dihydrochloride (Sigma-Aldrich) were added to the reaction mixture in PBS. Controls were prepared by omitting one component of the reaction (plasminogen, uPA, or substrate). The reaction was performed for 16 h at 37°C, and the absorbance of specific substrate degradation (OD_405nm_) was measured using a microplate reader.

## Results

### In silico analysis of protein conservation among leptospiral strains, identification of domains, and structural modeling

**Figure 1.**
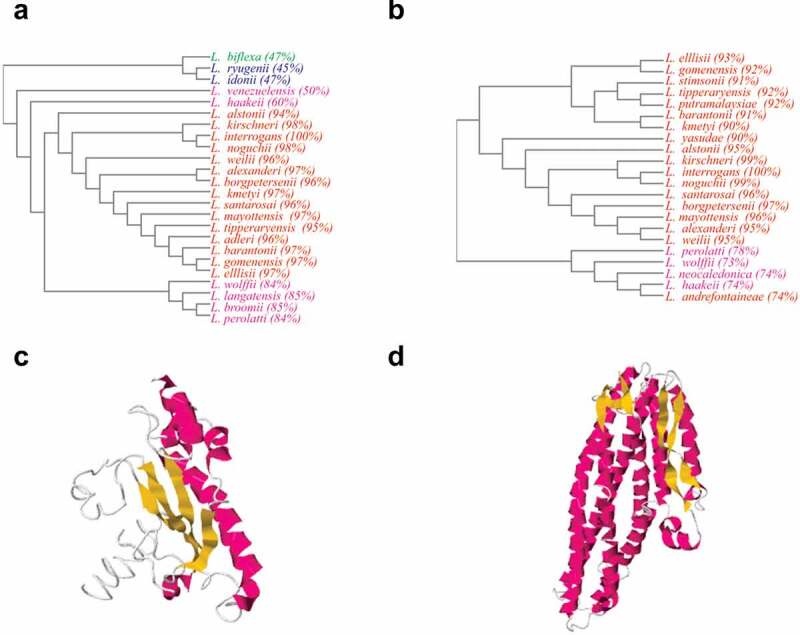
**Bioinformatic analysis of LipL21 and LipL41**. A and B: Phylograms obtained by Clustal Omega software, showing LipL21 and LipL41 sequences conservation among virulent leptospiral strains P1 (red) and P2 (purple), respectively; and saprophytic strains S1 (green) and S2 (blue) in distant branches. C and D: tertiary structure modeled by I-TASSER showing the alpha-helices (purple), beta-strands (yellow) and coil regions (write) of LipL21 and LipL41, respectively

LipL21 and LipL41 CDs were identified by *L. interrogans* serovar Copenhageni genome sequencing [30], and proteome analysis showed that both proteins are highly expressed [5]. Moreover, LipL21 and LipL41 are highly conserved, since both have been detected in several virulent strains [13,16]. Analysis of multiple sequence alignments by Clustal Omega confirmed that both LipL21 and LipL41 are conserved (Figures 1a and 1b, respectively), having more than 90% similarity among virulent strains (P1 and P2 subclades), and low or no similarity with saprophytic and intermediate strains (S1 and S2 clades). LipL21 showed 47% similarity with saprophytic *L. biflexa* (Figure 1a). However, when the antibody against LipL21 *of L. interrogans* was used in a total *L. biflexa* cell extract, no protein was detected [16], suggesting a distinct protein in saprophyte strains. LipL41 showed no similarity with intermediate or saprophytic strains (Figure 1b). Both proteins presented a proximity to *L. kirschneri* and *L. noguchii* by sharing the closest branch of the phylogenetic tree, and the strains are classified at the P1 subclade [31].

Analysis of domains using the NCBI, PFAM, and SMART webservers showed that LipL21 does not match with any conserved domain. LipL41 alignments showed two tetratricopeptide (TPR) repeats, which are involved in protein–protein interactions and multiprotein complex assemblies. Heme regulatory motifs (HRMs) were experimentally described at ^140^Cys-Ser and ^220^Cys-Pro of LipL41 [14]. The model of the tertiary structure for LipL21 (Figure 1c) showed a C-score of −3.71. When aligned against PDB using TM-align, (Template Modeling score was used as confidence value, considering the TM-score from [0,1]) ranging between 0.553 to 0.455, showed structural analogy with some enzymes, such as aldehyde dehydrogenase of *Desulfovibrio gigas* (PDB ID: 1SIJ), transferase of *Legionella pneumophila* and *Saccharomyces cerevisiae* calmodulin complex (PDB ID: 6OQQ), oxidorectase from *Thauera aromatica* (PDB ID: 1SB3), methyltransferase from *Escherichia coli* (PDB ID: 1MSK), unknown function protein from *Treponema pallidum* (PDB ID: 5JIR), and transport proteins from *Antheraea pernyi* and *Thermotoga maritima* (PDB ID: 3 GWJ and 3DIN). Modeling of LipL41 (Figure 1d, C-score of −1.42) and the results of alignment (TM-score ranging 0.891 to 0.574) revealed the analogy to some toxins of *Bacillus cereus, Xenorhabdus nematophila, Aeromonas hydrophila*, and *Vibrio cholerae* (PDB ID: 4K1P, 6EZV, 6W08A, 6GY6, 6GRJ, and 6W1W), membrane protein of *Yersinia enterocolitica* (PDB ID: 6EK7), and hemolysins of *E. coli* and *B. cereus* (PDB ID: 1QOY and 2NRJ). The alignments suggest that LipL21 can act as an enzyme or transport protein, having similarity with a protein from the spirochete *T. pallidum*, whereas LipL41 protein alignments show analogy to membrane proteins and virulence factors associated with toxins and hemolysins.

### Cloning and characterization of recombinant proteins

**Figure 2.**
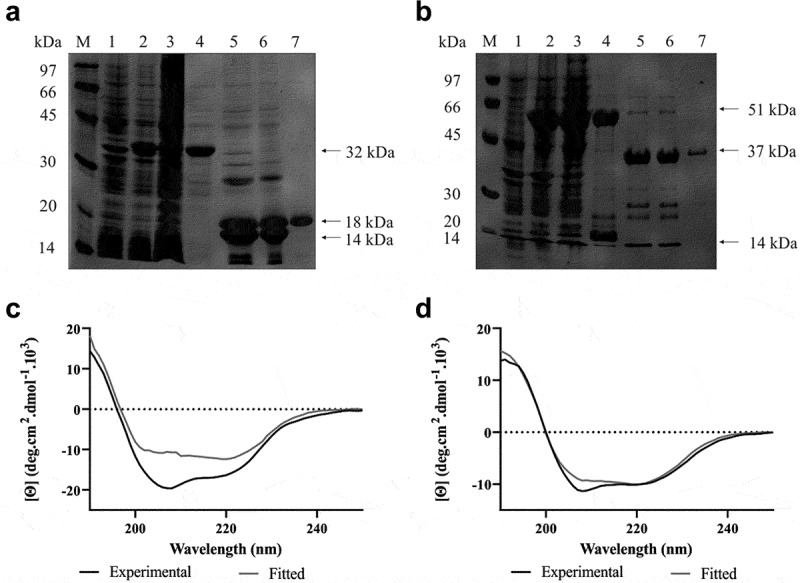
**Expression, purification and secondary structure analysis of recombinant LipL21 and LipL41**. In A (LipL21) and B (LipL41): 12% SDS-PAGE analysis of expression and purification. M: molecular mass protein marker, lane 1: non-induced *E. coli* cell lysate, lane 2: bacterial extract after induction with 1 mM IPTG, lane 3: soluble fraction, lane 4: elution of recombinant proteins, lane 5: cleavage of recombinant proteins by ULP-1 (SUMO protease), lane 6: dialyzed proteins, and lane 7: recovered recombinant proteins. The arrows show the predicted molecular weight of the recombinant protein with (32 and 51 kDa, for LipL21 and LipL41, respectively) and without (18 and 37 kDa, for LipL21 and LipL41) the fusion tag (approximately 14 kDa). In C (LipL21) and D (LipL41): evaluation of secondary structure by circular dichroism. Experimental measures were performed at 20°C in a spectropolarimeter recorded from 190 to 250 nm. Curves show the mean of 10 scans from experimental results obtained by dichroism (in black) and the fitted curve by the software BestSel (in gray)

The CDs of LipL21 (LIC10011) and LipL41 (LIC12966) were amplified by PCR without the signal peptide sequence, showing the expected size of 504 bp and 1,008 bp, respectively, and were cloned into the pET28a-SUMO vector [25]. We choose to use this cloning system in order to achieve the recombinant protein in its soluble form, avoiding the urea denaturing steps. Purification was performed from the soluble fraction using IMAC, and the eluted fractions were evaluated by SDS-PAGE. Protein expression and purification profiles obtained for LipL21 and LipL41 have been presented in Figures 2a and 2b, respectively. The final purification step (Figures 2a and 2b, lane 7) was successful for both proteins, with the amount of protein recovered from the purification of LipL21 being higher than that of LipL41. Following dialysis, the recombinant protein secondary structures were modeled using BeStSel software using the CD spectra. The curves of the experimental assay and the fitted curves obtained from the software are shown in Figures 2c and 2d. LipL21 structural analysis (Figure 2c) revealed 63.1% of alpha helix, 3.3% antiparallel, and 33.5% other structures, whereas LipL41 (Figure 2d) had 52.5% of alpha helix, 3.2% antiparallel, 1.5% turns, and 25.3% other structures. The theoretical value for LipL21 was 23% alpha helix, 39% antiparallel and 38% irregular. For LipL41, 68% was found to be alpha helix, 16% antiparallel and 16% irregular. Differences from theorical and experimental results can be due to the conditions used in the experimental assays, as the buffer ionic strength, pH, and temperature.

### Interaction of recombinant proteins with purified host components

**Figure 3.**
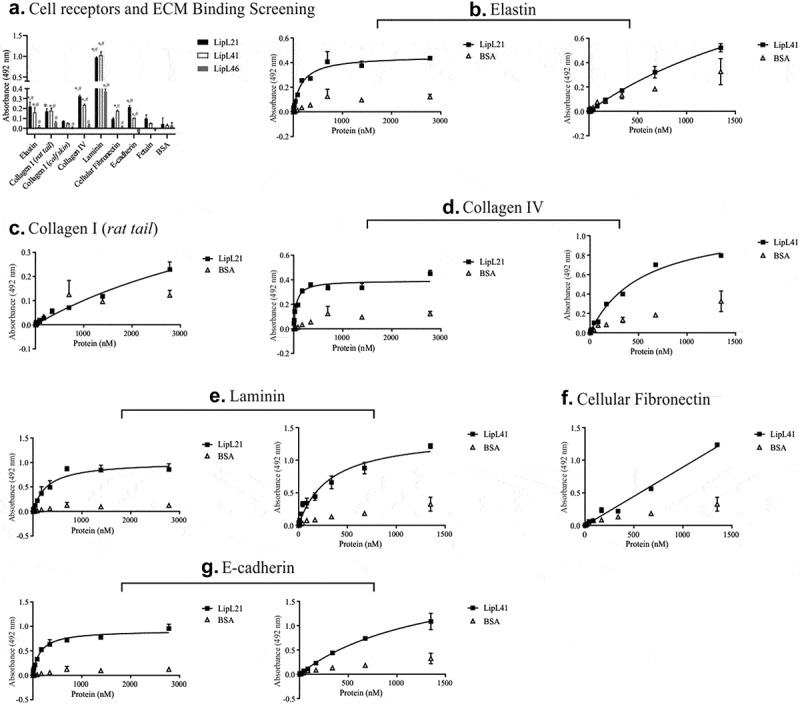
**Binding of recombinant proteins to the cell receptors and ECM components**. 1 µg of elastin, collagen type I (*rat tail* and *calfskin*), laminin, cellular fibronectin, and e-cadherin was immobilized in 96 wells ELISA plates. Fetuin and BSA were used as negative controls. Recombinant proteins were allowed to interact with the components for 2 hours. LipL46 was used as protein control. Then, polyclonal antibodies against each recombinant protein were added and the ligation detection was performed using anti-mouse IgG-peroxidase (1:5,000). The ligation of the recombinant proteins was compared to the negative controls by the two-tailed *t*-test (* represents BSA and # represents Fetuin, p < 0.05). The dose-dependent curves were fitted using the GraphPad Prism software. A: Binding to the extracellular matrix (LipL21 is shown in black bars and LipL41 is represented by white bars). B to G: evaluation of binding to the ECM components with the increasing of recombinant proteins concentration. B: elastin, C: collagen I (*rat tail*), D: collagen IV, E: laminin, F: cellular fibronectin and G: E-cadherin. Bars and points represent the mean absorbance at 492 nm ± SD of three replicates

Pathogen adherence to the host is one of the main characteristics for maintaining colonization and infection. Therefore, we examined the ability of recombinant LipL21 and LipL41 to mediate the interaction with host components. ECM proteins, plasma proteins, GAGs, and the control proteins BSA and fetuin were immobilized on 96-well plates, followed by the evaluation of LipL21 and LipL41 attachment using antibodies against the recombinant proteins. Both proteins showed similar and broad-spectrum binding to ECM molecules (Figure 3). Statistically significant binding of LipL21 and LipL41 was observed for elastin, collagen I from *rat tail*, collagen IV, laminin, and E-cadherin (Figure 3a). LipL41 also bound to cellular fibronectin, although none of the proteins interacted with collagen I from calf skin. These interactions were confirmed on a quantitative basis. Dose dependent binding was observed when 0–6 µM LipL21 and 0–1.5 µM LipL41 were allowed to interact with elastin (Figure 3b), collagen I from *rat tail* (Figure 3c), collagen IV (Figure 3d), laminin (Figure 3e), cellular fibronectin (figure 3f), and E-cadherin (Figure 3g). Binding saturation was observed only for LipL21 with elastin (Figure 3b, dissociation constant [K*_D_*] = 171.4 ± 26.8 nM), collagen IV (Figure 3d, K*_D_* = 53.9 ± 9.8 nM), laminin (Figure 3e, K*_D_* = 287 ± 58.9 nM), and E-cadherin (Figure 3g, K*_D_* = 146.6 ± 18.6 nM).

**Figure 4.**
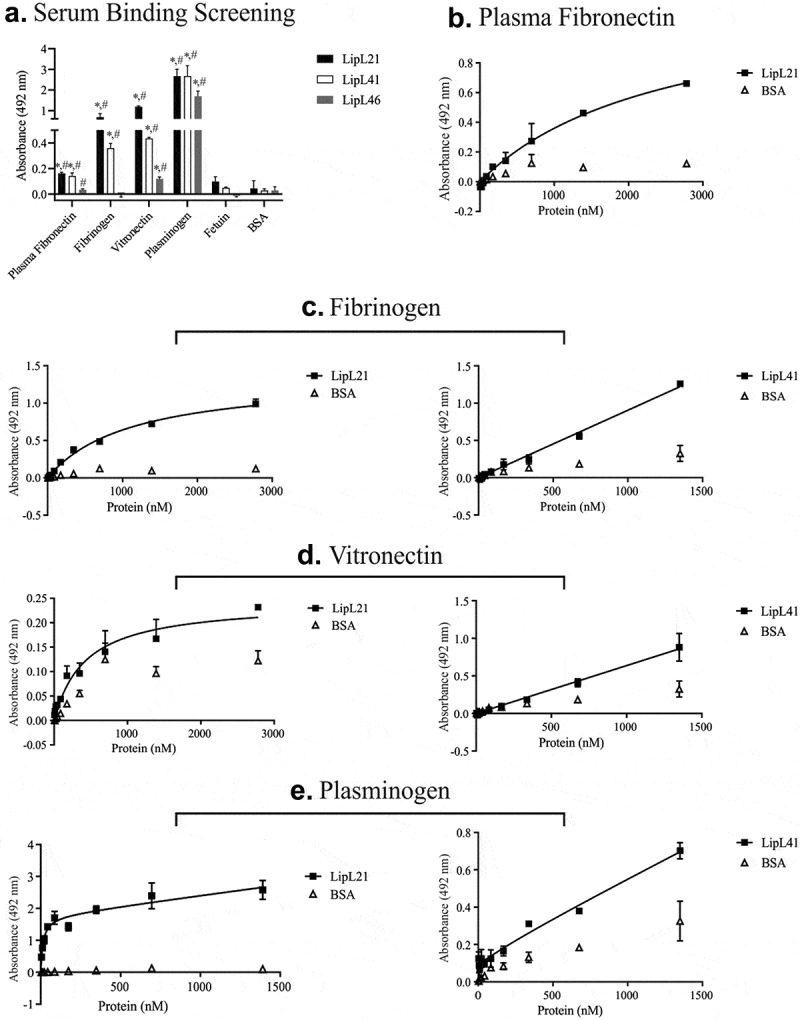
**Binding of recombinant proteins to the plasma components**. 1 µg of plasma fibronectin, fibrinogen, vitronectin, and plasminogen as plasma components were immobilized in 96 wells ELISA plates. Fetuin and BSA were used as negative controls. Recombinant proteins were allowed to interact with immobilized components for 2 hours. LipL46 was used as protein control. Polyclonal antibodies against each recombinant protein were added and ligation detection was performed using anti-mouse IgG-peroxidase (1:5,000). The binding of the recombinant proteins was compared to the negative controls by the two-tailed *t*-test (* represents BSA and # represents Fetuin, p < 0.05). The dose-dependent curves were fitted using the GraphPad Prism software. A: binding to the plasma components (LipL21 is shown in black bars and LipL41 is represented by white bars). B to E: evaluation of binding to the plasma components with the increasing of recombinant proteins concentration. B: plasma fibronectin, C: fibrinogen, D: vitronectin and E: plasminogen. Bars and points represent the mean absorbance at 492 nm ± SD of three replicates

LipL21 and LipL41 interacted with all plasma proteins tested, showing dose-dependent binding for all proteins except plasma fibronectin (Figure 4a). Only LipL21 interacted in a dose dependent manner with plasma fibronectin (Figure 4b). Both proteins interacted with fibrinogen in a dose dependent fashion (Figure 4c). LipL21 showed high binding affinity to vitronectin; BSA, used as a control, also demonstrated strong dose dependent binding (Figure 4d). Increase in BSA binding was also observed for the LipL41 interactions. Thus, LipL21 exhibited greater binding affinity to all these components as compared to LipL41.

**Figure 5.**
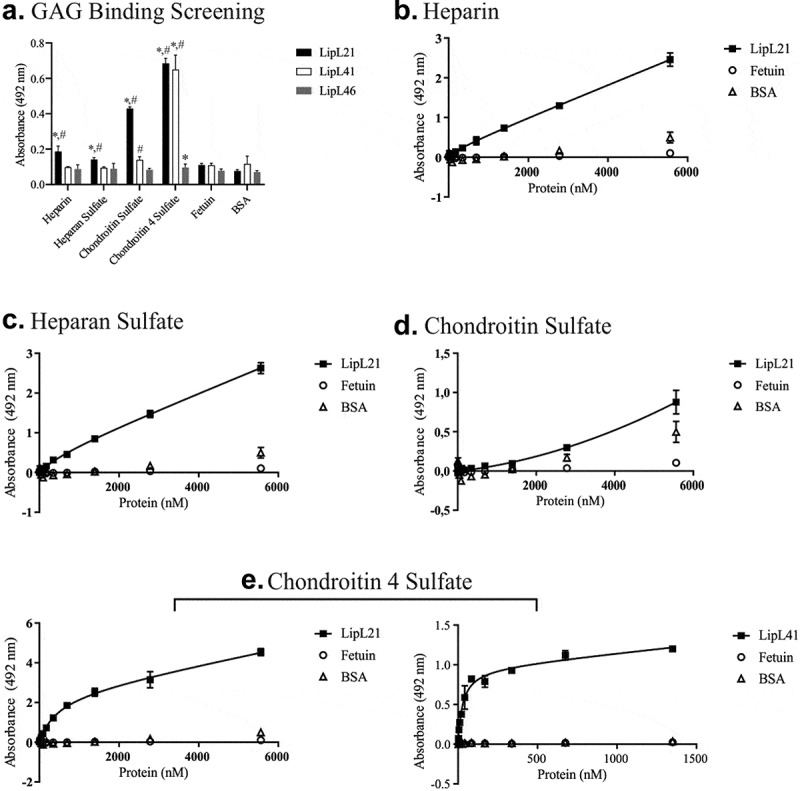
**Binding of recombinant proteins to GAGs**. ELISA plates (96 wells) were coated with 100 µg heparin, chondroitin sulfate, and chondroitin 4 sulfate, and 1 µg of heparan sulfate. Fetuin and BSA were used as negative controls. Recombinant proteins were incubated for 2 hours at 37°C. LipL46 was used as protein control. The binding mixture was fixed with 2% paraformaldehyde, followed by an incubation of 2% glycine in PBS for 30 min at RT. Polyclonal antibodies against each recombinant protein were incubated for 1 hour at 37°C. Ligation detection was performed using anti-mouse IgG-peroxidase (1:5,000). The binding of the recombinant proteins was compared to the negative controls by the two-tailed *t*-test (* represents BSA and # represents Fetuin, p < 0.05). The dose-dependent curves were fitted using the GraphPad Prism software. A: Binding to the glycosaminoglycans (LipL21 is shown in black bars and LipL41 is represented by white bars). B to E: evaluation of binding to the glycosanimoglycans with the increasing of recombinant proteins concentration. B: heparin, C: heparan sulfate, D: chondroitin sulfate and E: chondroitin 4 sulfate. Bars and points represent the mean absorbance at 492 nm ± SD of three replicates

LipL21 also showed a broad-range binding profile with GAGs. As shown in Figure 5a, LipL21 interacted with heparin, heparan sulfate, chondroitin sulfate, and chondroitin 4 sulfate, whereas LipL41 showed a statistically significant difference only when interacting with chondroitin 4. Dose dependent binding confirmed the interaction with all these components, though a saturation level was not reached (Figure 5b–e). Although the interaction of LipL41 and chondroitin 4 sulfate did not reach the saturation level (Figure 5e), the calculated K*_D_* value suggests high binding affinity (K*_D_* = 27.94 ± 8.1 nM).

The curves were fitted using the software GraphPad Prism using non-linear regression, which calculated the K*_D_* of ligation, considering binding saturation (Table 1).

**Table 1.** Dissociation constants (K*_D_*) of the recombinant proteins binding to extracellular matrix, plasma and GAG components (nM)

Host localization	Component	LipL21	LipL41
Cell receptors and Extracellular matrix	Elastin	171.4 ± 26.8	2720 ± 620.5
	Collagen I (*rat tail*)	6136 ± 2601	
	Collagen IV	53.9 ± 9.8	536.6 ± 95.6
	Laminin	287 ± 58.9	311.6 ± 64.4
	Cellular Fibronectin		-
	E-cadherin	146.6 ± 18.6	1375 ± 241.1
Plasma	Plasma Fibronectin	2545 ± 692.7	
	Fibrinogen	1119 ± 117.3	-
	Vitronectin	424.4 ± 95.3	-
	Plasminogen	13.85 ± 11.3	-
GAG	Heparin	-	
	Heparan Sulfate	724.9 ± 485.4	
	Chondroitin Sulfate	-	
	Chondroitin 4 Sulfate	437.4 ± 112.2	27.94 ± 8.1

### Inhibition of fibrin clot formation by recombinant proteins

**Figure 6.**
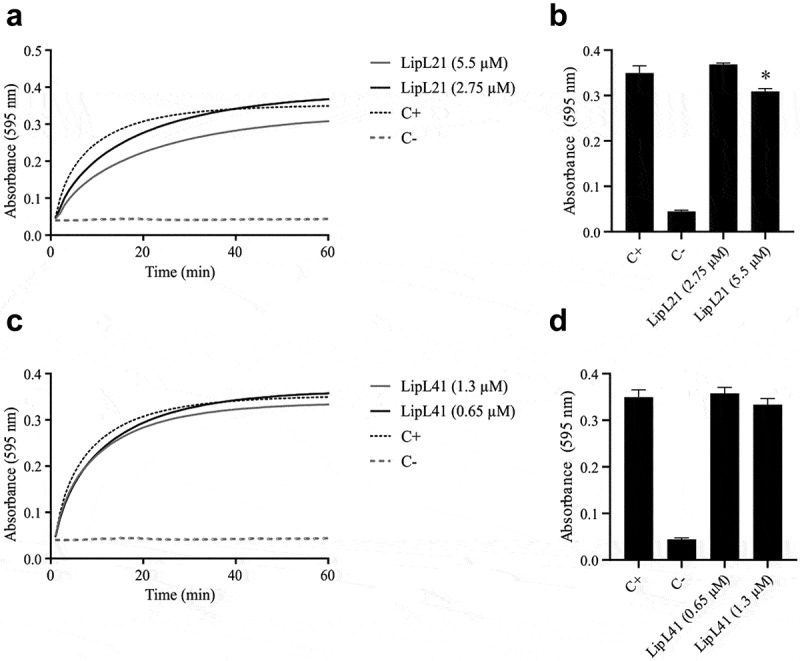
**Fibrin clotting inhibition assay**. The recombinant proteins were incubated with fibrinogen (1 mg/mL) for 2 hours at 37°C. Then, 10 µL of thrombin (10 U/mL) were added to reaction mixture. The fibrin clot measurements were taken every 1 min for until 60 min. The positive control is represented by the co-incubation of thrombin and fibrinogen and negative control is the reading of thrombin sample. In A: kinetics of fibrin clot formation and B: the last measurement of kinetic to show statistical significance (two-tailed *t*-test, p < 0.05) for LipL21 (a and b) and for LipL41 (c and d). Each point represents the mean absorbance at 595 nm ± SD of three replicates

The coagulation cascade involves the activation of prothrombin to thrombin, which in turn catalyses the reaction of soluble fibrinogen to insoluble fibrin. Fibrin clot formation stops the bleeding of damaged blood vessels, subsequently inhibiting hemorrhage [32]. It has been demonstrated that during leptospirosis, leptospires bind to fibrinogen and decrease fibrin clot formation, helping bacterial dissemination [33,34]. As both LipL21 and LipL41 bind to fibrinogen, we investigated if these proteins could mediate the inhibition of fibrin clot formation. Measurements were performed in the presence of two concentrations of proteins, the higher concentration achieved for each protein after purification, and temporal changes were recorded. As shown in Figure 6a, the kinetics of fibrin clot formation show that only LipL21, used at the highest concentration, was able to decrease fibrin clot formation by a thrombin-catalyzed reaction. Data presented in Figure 6b indicate a statistically significant difference only for LipL21. Although LipL41 showed a tendency to reduce fibrin clotting, it was not confirmed statistically.

### Characterization of recombinant protein interaction with plasminogen

Plasminogen is activated by proteolysis, and its structure contains five regions of approximately 80 amino acids linked by a disulfide bond. These regions are called kringle domains. Their function is important as they can mediate the binding to pathogens and mammalian cell surface [35]. It was reported that pathogenic leptospires can bind to plasminogen by the kringle domains, and the binding of leptospires to plasminogen was decreased when co-incubated with lysine analogues, such as ACA, suggesting that those interactions occur via lysine residues. Furthermore, it has been shown that leptospires were able to convert plasminogen to active plasmin in the presence of exogenous uPA [36].

**Figure 7.**
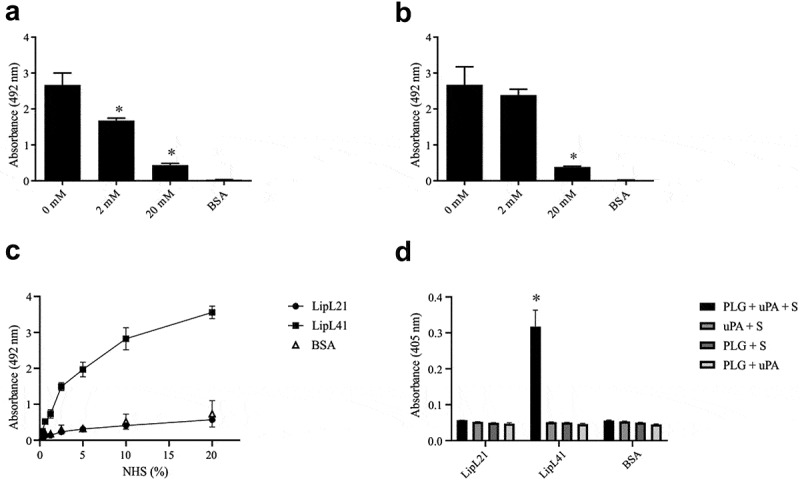
**Characterization of the recombinant proteins interactions with plasminogen. In A (LipL21) and B (LipL41): Amino caproic acid effect on recombinant proteins binding to plasminogen**. Ninety-six wells ELISA plates were coated with 1 µg of plasminogen and BSA, used as the negative control. Recombinant proteins were co-incubated with (0, 2, and 20 mM) amino caproic acid for 2 hours and ligation detection was performed as described above. The ligation of the recombinant proteins to plasminogen was compared to no-inhibition treatment by the two-tailed *t*-test (p < 0.05). Bars and points represent the mean absorbance at 492 nm ± SD of three replicates. **C: Recruitment of plasminogen by the recombinant proteins from NHS**. Ninety-six wells ELISA plates were coated with 1 µg of recombinant proteins and BSA, used as the negative control. NHS was added to wells from 0 to 20%, followed by addition of anti-plasminogen. The ligation of the recombinant proteins to plasminogen was compared to BSA by the two-tailed *t*-test (p < 0.05). **D: Enzymatic assay of plasminogen converting to plasmin in the presence of uPA**. Ninety-six wells ELISA plates were coated with 1 µg of recombinant proteins and BSA used as the negative control. Then, Plasminogen (1 µg) was added to wells for 2 hours, followed by the addition of uPA and/or plasmin substrate for 16 hours at 37°C. The conversion of plasminogen to plasmin was compared to BSA by the two-tailed *t*-test (p < 0.05). Bars represent the mean absorbance at 405 nm ± SD of three replicates relative to substrate degradation

This encouraged us to examine whether the LipL21 and LipL41 interact with plasminogen via lysine residues. The results shown in Figures 7a and 7b suggest the involvement of lysine residues, because the binding of both proteins decreased when incubated with the competitive inhibitor ACA. A low concentration (2 mM) of ACA was sufficient to reduce the binding of LipL21. In contrast, 20 mM ACA promoted the inhibition of LipL41 binding.

The proteins were also allowed to interact with normal human serum (NHS) to verify whether LipL21 and LipL41 are able to recruit plasminogen directly from serum. As shown in Figure 7c, only LipL41 was able to recruit plasminogen from NHS. Although LipL21 showed a higher affinity to plasminogen than LipL41, it was not able to perform such a function. Moreover, only LipL41 showed the ability to convert plasminogen into active plasmin in the presence of uPA (Figure 7d).

## Discussion

LipL21 and LipL41 are considered major outer membrane proteins of pathogenic *Leptospira* spp. It has been reported that LipL21 and LipL41 exhibit immunogenic potential for vaccine formulations individually, in the chimeric form, or in combination with other proteins. Moreover, their use as diagnostic molecules has shown high accuracy [37–42]. LipL21 can contribute to leptospiral virulence as it resists peptidoglycan degradation, as well as NOD1 and NOD2 recognition [43]. Moreover, LipL21 could act as a potent myeloperoxidase inhibitor, an enzyme stored in neutrophils [44]. Upon interaction with a host, LipL41, described as a hemin-binding protein, is able to expresses seven isoforms, which could be related to its virulence [14,45].

The pathogenic interactions with host components represent important steps in adhesion, invasion, and evasion of the immune system. Several pathogens are able to express adhesins, which have the ability to interact with the ECM. The ECM is present around the cells, and its main function is to provide a structure for cell–cell adhesion and a support for tissues, cell migration, and signaling, in addition to acting as a barrier that protects the tissue from pathogens [46]. The main components that comprise the ECM are fibrous proteins, such as collagen, laminin, fibronectin, vitronectin, elastin, and proteoglycans, the latter being constituted by a core protein and GAG chains [47]. The interaction of pathogens with the ECM can promote degradation of its components or increase the permeability of the tissue by disrupting cell–cell interactions, and thus, facilitate the penetration and dissemination of pathogen [48,49].

In previous studies addressing adhesion, the binding of pathogenic *Leptospira* to fibronectin, collagens I and IV, and laminin was demonstrated [50]. Subsequently, several leptospiral proteins have been identified as adhesins [51–59]. Many proteins have the ability to bind with laminin, whereas a few have been identified as ligands of elastin, collagen, and E-cadherin. Here, we showed that both LipL21 and LipL41 were able to adhere to all the ECM components tested, except for collagen I from calf skin. As an interaction was observed for collagen I from rat tail, it is suggested that the binding affinity between components can change depending on their origin. Both proteins showed dose dependent binding; however, LipL21 likely possessed a higher affinity for interaction, because the K*_D_* values obtained were lower than those previously reported [17]. For instance, LipL32 showed a K*_D_* of 599 ± 12 nM for collagen IV [60], whereas the interaction between OmpL1 and laminin yielded a K*_D_* of 2099 ± 871 nM [61]. OmpL37 showed a K*_D_* of 104 ± 19 nM for aortic elastin [62], whereas rLIC11711 and rLIC10831 presented K*_D_* values of 3.82 ± 0.21 µM and 2.3 ± 0.3 µM, respectively, for E-cadherin [63,64]. As LipL21 and LipL41 showed broad spectrum ECM binding, it is likely that both proteins contribute significantly during the initial steps of leptospiral infection.

During invasion through the skin and mucosa, leptospires reach the bloodstream by crossing the endothelium, and remain there for approximately seven days, until clearance by antibody production. Otherwise, they proceed and colonize the renal proximal tubules in the luminal region [65]. After passing through the epithelia (skin and mucosa) and endothelia (blood vessels), leptospires can interact with plasma molecules, components of the complement system, and glycoproteins related to coagulation and fibrinolysis [66]. Several virulent leptospiral strains are able to recruit vitronectin from normal human plasma [67]. Plasma vitronectin can interact with the plasminogen activation complex and regulate the terminal pathway of complement activation [68]. Thus, *Leptospira* can interfere at specific points of these pathways, favoring its survival. Like LcpA, rLIC11711, and rLIC13259, the recombinant proteins LipL21 and LipL41 were also able to mediate the interaction with vitronectin [64,67,69]. Moreover, both LcpA and rLIC13259 can interfere with the terminal complement pathway, preventing membrane attack complex (MAC) deposition on the bacterial surface [67,69]. The interaction of LipL21 and LipL41 with the terminal complement pathway molecules is currently being addressed.

Fibrinogen is a glycoprotein found at high concentrations in plasma, mainly in association with the coagulation cascade by the conversion to fibrin, which stabilizes the blood clot [70]. *Leptospira* can inhibit the fibrin clot formation as a virulence strategy. Some surface proteins have already been identified as being capable of mediating such an interaction [34]. Here, we showed that despite the interaction of LipL41 and LipL21 with fibrinogen, only LipL21 showed a decrease in fibrin clot formation. However, the reduction was minimal compared with other proteins that are able to deter fibrin clotting [34]. It is still unclear why all proteins do not have the inhibitory ability; however, it is not exclusive for LipL41. Lsa16 was not able to inhibit fibrin clot formation as well [71]. The capture of plasminogen on their surface and its conversion to plasmin has contributed to invasion and the evasion of host immune system by pathogens [72]. It has been demonstrated that pathogenic leptospires can generate plasmin, which can degrade laminin and fibrinogen, and decrease C3b and IgG depositions [34,36,73]. Several leptospiral proteins have been shown to interact with plasminogen and convert it to plasmin in the presence of plasminogen-activator. Although, LipL21 and LipL41 interact with plasminogen in a dose-dependent manner, plasmin was only observed when LipL41 is bound to plasminogen, suggesting that the enzymatic activation sites by urokinase were possibly inaccessible in the presence of LipL21. Further, LipL21 could not capture plasminogen from NHS. As LipL21 showed broad spectrum binding to many components, overlapping regions of interaction were likely competing for LipL21 binding.

GAGs are expressed by nucleated cells in the ECM, and interact with the other molecules of the ECM to stabilize cell–cell adhesion [74]. Pathogens such as *Neisseria gonorrhoeae, Bordetella pertussis, Mycobacteria* spp., *Listeria monocytogenes, E. coli*, and the spirochete *Borrelia burgdorferi* express surface proteins that can attach to GAGs, suggesting the importance of GAG binding for host adhesion and invasion [75,76]. A few studies have explored the interaction of pathogenic leptospires to GAGs, and only OmpL1 has been identified as a GAG binding protein [19,77]. Here, we showed that LipL21 has a binding spectrum similar to OmpL1, because it was able to interact with chondroitin sulfates, heparin, and heparan sulfate; LipL41 interacted only with chondroitin 4 sulfate. Our results corroborate the data obtained previously [77], which show that leptospires binding to GAGs interact more efficiently with chondroitin sulfate than with heparan sulfate.

In conclusion, our findings show that LipL21 and LipL41 are multifunctional adhesins with the capacity of binding with several host components. It is anticipated that the ability of LipL21 and LipL41 to adhere to the ECM and interact with plasma proteins may contribute to successful *Leptospira* colonization by participating in multiple steps of invasion. Indeed, the presence of proteins with a broad-spectrum binding profile is not unique of *Leptospira*, and is probably part of the bacterial strategy to escape the host’s barriers [78–82]. To the best of our knowledge, LipL21 and LipL41 are the first leptospiral lipoproteins reported to possess the ability to interact with GAG. Further investigations on leptospiral molecular mechanisms in immune evasion and mammalian cell adherence by these proteins, as well as, leptospiral mutants, remain to be ascertained.

## Data Availability

The manuscript data will be available after acceptance. Our data will be freely available upon request from our laboratory. Other cited data are referenced and available at PuBMed.

## Abbreviations

Small ubiquitin-like modifier (SUMO); glycosaminoglycans (GAGs)
